# Colonic obstruction secondary to lithobezoar in a child

**DOI:** 10.11604/pamj.2022.42.272.36514

**Published:** 2022-08-11

**Authors:** Ahmed El Mouloua

**Affiliations:** 1 Pediatric Surgery Unit, Sidi Mohamed Ben Abdellah Hospital, Essaouira, Morocco

**Keywords:** Lithobezoar, children, colonic obstruction

## Image in medicine

A nine-year-old girl with no history of abdominal surgery was presented to the emergency department with a seven-day-lasting abdominal pain, yellow vomiting and constipation; the stool was brownish mixed with small stones. There was no history of fever or similar episodes. Her development history revealed multiple episodes of earth and stone-eating since her 3 years. The physical examination revealed a well-developed child with stable vital signs, the abdomen was slightly distended and tense in its lower part. There was a palpable lump in the hypogastric area and in left iliac fossa. Rectal examination demonstrated a rectal ampulla full of small stones. Abdominal X-ray showed gravel inside the large bowel and rectum with a distended transverse and left colic angle. Manual evacuation and colonic lavage were done and repeated twice daily for three days. On the fourth day, an abdominal X-ray showed clearance of all stones from the colon and no signs of colonic obstruction or perforation.

**Figure 1:**
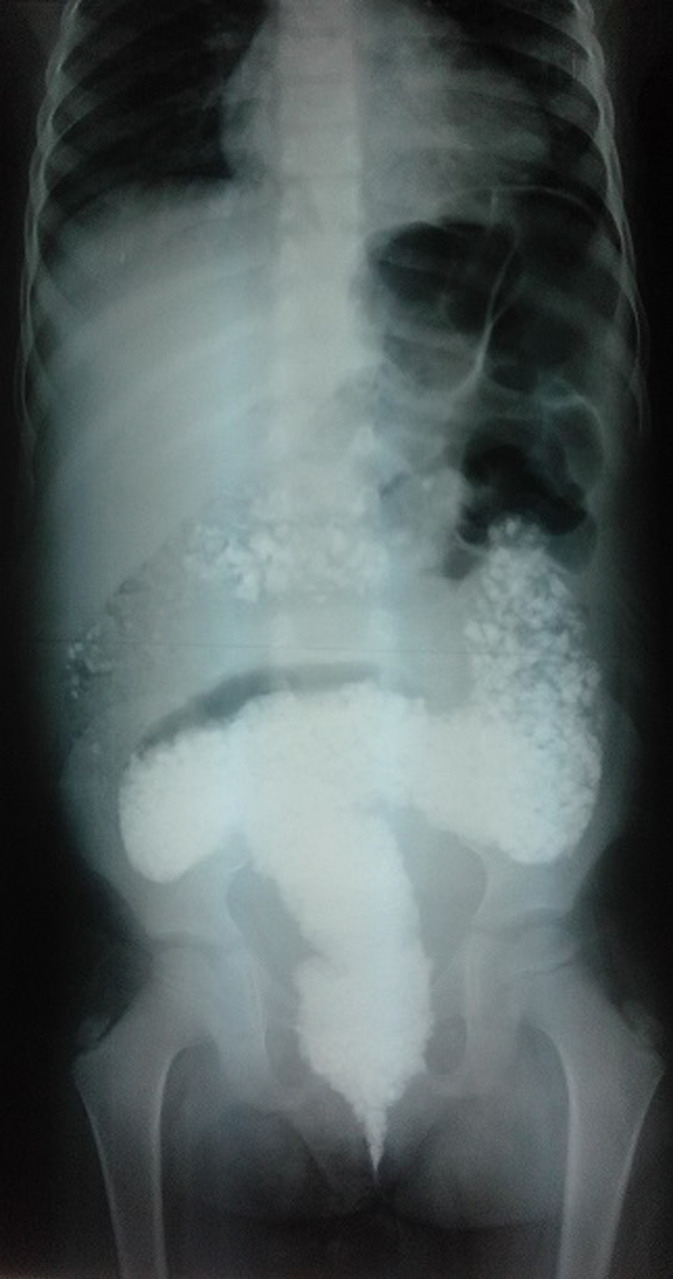
X-ray of the abdomen showing gravel inside the large bowel and rectum

